# MRI-based in vivo detection of coronary microvascular dysfunction before alterations in cardiac function induced by short-term high-fat diet in mice

**DOI:** 10.1038/s41598-021-98401-1

**Published:** 2021-09-23

**Authors:** Grzegorz Kwiatkowski, Anna Bar, Agnieszka Jasztal, Stefan Chłopicki

**Affiliations:** 1 Jagiellonian Centre for Experimental Therapeutics (JCET), Jagiellonian University, ul. Bobrzynskiego 14, 30-348 Kraków, Poland; 2 Chair of Pharmacology, Faculty of Medicine, Jagiellonian University Medical College, Grzegorzecka 16, 31-531 Kraków, Poland

**Keywords:** Biomarkers, Cardiology, Diseases, Medical research

## Abstract

Endothelial dysfunction is one of the hallmarks of vascular abnormalities in metabolic diseases and has been repeatedly demonstrated in coronary and peripheral circulation in mice fed high-fat diet (HFD), particularly after long-term HFD. However, the temporal relationship between development of coronary microvascular endothelial dysfunction and deterioration in diastolic and systolic cardiac function after short-term feeding with HFD has not yet been studied. This study aimed to correlate the changes in coronary microvascular endothelial function and global cardiac performance indices in vivo after short-term feeding with HFD in mice. Short-term feeding with a HFD (60% fat + 1% cholesterol) resulted in severely impaired coronary microvascular function, as evidenced by the diminished effect of nitric oxide synthase inhibition (by L-NAME) assessed using T_1_ mapping via in vivo MRI. Deterioration of coronary microvascular function was detected as early as after 7 days of HFD and further declined after 8 weeks on a HFD. HFD-induced coronary microvascular dysfunction was not associated with impaired myocardial capillary density and was present before systemic insulin resistance assessed by a glucose tolerance test. Basal coronary flow and coronary reserve, as assessed using the A_2A_ adenosine receptor agonist regadenoson, were also not altered in HFD-fed mice. Histological analysis did not reveal cardiomyocyte hypertrophy or fibrosis. Increased lipid accumulation in cardiomyocytes was detected as early as after 7 days of HFD and remained at a similar level at 8 weeks on a HFD. Multiparametric cardiac MRI revealed a reduction in systolic heart function, including decreased ejection rate, increased end-systolic volume and decreased myocardial strain in diastole with impaired ejection fraction, but not until 4 weeks of HFD. Short-term feeding with HFD resulted in early endothelial dysfunction in coronary microcirculation that preceded alteration in cardiac function and systemic insulin resistance.

## Introduction

Coronary macrocirculation, consisting of the epicardial coronary arteries, has mostly a conductance role and, as such, exhibits little resistance to coronary flow when not obstructed. In contrast, coronary microcirculation of pre-arterioles and arterioles encloses the majority of the blood within the coronary circulation and comprises most of the resistance circuit of the heart. To adapt to varying mechanical workloads, the heart relies on extensive vasomotor regulation of both macro- and microcirculation^1^. Consequently, any mismatch between vasomotor augmentation of coronary microcirculation and increased oxygen demand may lead to diminished blood supply to the myocardium and, in turn, to ischaemic conditions^2,3^.

Often synonymous with coronary artery disease (CAD), stenosis of epicardial coronary arteries has long been recognized as a risk factor for ischaemic heart disease (IHD). However, an emerging body of evidence suggests that coronary microvascular dysfunction (CMD) is an important contributor to myocardial ischaemia in both the presence and absence of epicardial coronary atherosclerosis^4^. Furthermore, CMD is commonly present in patients with metabolic derangements and has been reported in various models of metabolic syndrome in animals^5^.

There is overwhelming evidence that endothelial dysfunction in large conduit vessels as well as in the microcirculation is a hallmark of vascular dysfunction associated with obesity and insulin resistance. Several studies have demonstrated compromised endothelial function in conduit vessels in subjects with type I diabetes or type II diabetes, pre-diabetic patients and healthy volunteers exposed to prolonged glucose administration^6^. Standard protocols to assess systemic endothelial dysfunction in humans are based on changes in brachial artery vasomotor function during flow-mediated vasodilation (FMD)^7^.

The classical method to assess endothelial function in experimental models is based on ex vivo measurements of endothelial-dependent responses in large arteries or small arteries, as well as in isolated perfused heart, and all of these approaches were used to study impaired endothelial function caused by metabolic derangements induced by diabetes or by high fat diet (HFD)^8–10^.

Recently, an in vivo MRI method was adopted to characterise endothelial function in large or conduit-type of vessels^11^ and has proven reliable to detect early endothelial dysfunction in the aorta induced by short-term feeding with HFD^12^ as well as to detect endothelial dysfunction in vivo in large arteries in various murine models^13–15^.

In contrast to endothelial testing of large vessels, quantitative assessment of coronary microvascular function in vivo is challenging due to technical difficulties with directly imaging microvascular beds. In particular in small experimental animals, probing of the coronary endothelial status requires invasive Doppler wire catheterisation, not easily feasible in small animals. Instead, myocardial blood flow quantification under rest and flow-increased conditions is used as a surrogate for coronary microvascular status^16,17^. Recently, Cui et al.^18^ demonstrated that dynamic cardiac T_1_ MRI mapping with subsequent endothelial nitric oxide synthase (eNOS) inhibition with Nω-Nitro-l-arginine methyl ester (L-NAME) can provide direct quantification of nitric oxide (NO)-dependent function, one of the key mediators regulating endothelial function. By rapidly depleting the local vessel NO production, endothelial permeability was altered, leading to changes in water and protein efflux through the vessel lumens^19–21^. The acute changes in tissue water content were directly probed in vivo via T_1_ relaxation mapping, and this effect was proportional to eNOS function^18^. In this study, taking advantage of the MRI-based method proposed by Cui et al.^18^, as well as other complementary methods, we comprehensively evaluated the temporal relationship between the alteration in coronary microcirculation status and deterioration in cardiac function in response to short-term feeding with an HFD in mice. Changes in coronary microvascular NO-dependent function and global LV function and function of the left ventricle were assessed at several time points during a HFD, from 3 days to 8 weeks. Furthermore, the coronary blood flow reserve was assessed using Doppler flow velocity mapping under basal and hyperaemic conditions. Using this experimental approach, we demonstrated for the first time to our knowledge that as few as 7 days of HFD resulted in endothelial dysfunction in coronary microcirculation that occurred prior to alterations in cardiac function and systemic insulin resistance in HFD-fed mice.

## Results

### Insulin resistance and body mass change in mice fed a high-fat diet for 1–8 weeks

**Figure 1:**
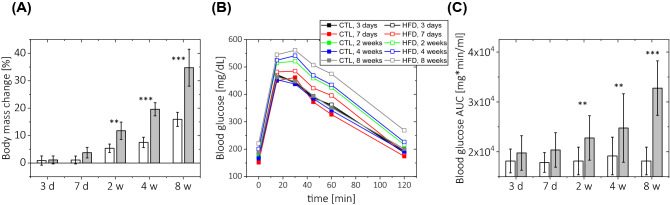
Effect of a high-fat diet on body mass and insulin resistance. (**A**) Body mass change (expressed as percentage of pre-diet body mass) throughout the diet duration. (**B**) Blood glucose levels measured at 0, 15, 30, 45, 60 and 120 min after glucose administration (2 g/kg *i.p.* (**C**) Area under the blood glucose level curves (AUC) shown in (**B**). The following number of animals was used: measurements at diet time *t* = 3 days, n_CTL_ = 5, n_HFD_ = 6; *t* = 7 days n_CTL_ = 5, n_HFD_ = 8;* t* = 2 weeks n_CTL_ = 6, n_HFD_ = 9;* t* = 4 weeks n_CTL_ = 8, n_HFD_ = 8; *t* = 8 weeks n_CTL_ = 5, n_HFD_ = 7.

As shown in Fig. 1, there were no significant changes in either body mass or results on the glucose tolerance test after 3 and 7 days on a HFD. However, body mass progressively increased starting from 14 days on a HFD, attaining maximum values of body mass increase after 8 weeks of HFD feeding, as shown in Fig. 1A. Parallel to a body mass change, significant insulin resistance was found starting at 14 days of HFD, but not earlier (i.e. after 3 or 7 days on a HFD). After 14 days of HFD a twofold increase in total glucose blood levels (AUC) following glucose infusion was found and at t = 8 weeks of HFD, AUC increased almost by threefolds, as shown in Fig. 1B,C.

### Coronary microvascular dysfunction in mice fed a high-fat diet for 1–8 weeks

**Figure 2:**
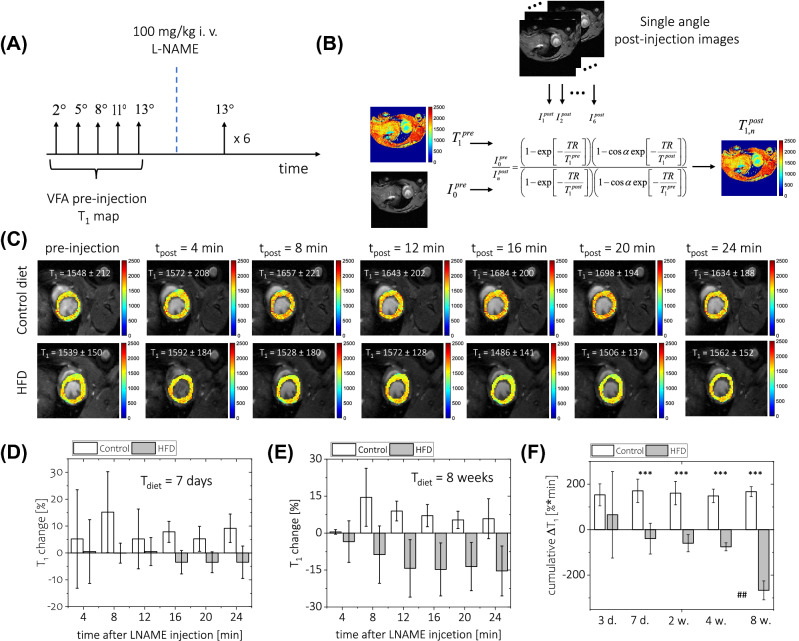
Assessment of endothelial dysfunction with NOS inhibition and in vivo T_1_ mapping. (**A**) Overview of the experimental protocol timing. A variable flip angle T_1_ map was obtained using five different RF excitation angles and used as a baseline T_1_ value. The baseline measurements were followed by 100 mg/kg *i.v.* L-NAME injection, and six continuous post-injection scans with the same RF excitation angle were recorded immediately after injection. The post-injection T_1_ maps were calculated using the signal changes in post-/pre-injection images and the pre-injection T_1_ map. (**C**) Examples of pre-/post-injection T_1_ maps (coloured scale, expressed in ms) overlaid on anatomical images (greyscale). Time course of changes in T_1_ values (expressed as percentage of baseline value) after L-NAME injection was calculated as an average over the whole myocardium and obtained after 7 days (**D**) and 8 weeks (**E**) on a diet. (**F**) The area under the curve of T_1_ change − time curves (cumulative ΔT_1_), ^*^ indicates comparison between control and HFD groups at given diet duration time whereas ^#^ indicates comparison within HFD group. For data in (**D**–**F**), results for control animals are given in white and data for high-fat diet-fed animals are given in grey. The following number of animals was used: measurements at diet time *t* = 3 days, n_CTL_ = 5, n_HFD_ = 6; *t* = 7 days n_CTL_ = 5, n_HFD_ = 8;* t* = 2 weeks n_CTL_ = 6, n_HFD_ = 9;* t* = 4 weeks n_CTL_ = 8, n_HFD_ = 8; *t* = 8 weeks n_CTL_ = 5, n_HFD_ = 7.

Intravenous injection of 100 mg/kg of L-NAME resulted in significant changes in myocardial T_1_ time, as shown in Fig. 2C. In a group of animals fed with a standard diet, eNOS inhibition with L-NAME increased T_1_ relaxation time, with the maximum change occurring at 8 min post-injection and at a value of approximately 15% of baseline T_1_ time (Fig. 2D,E). A similar time course was observed across all groups of control animals. In contrast, in animals fed with a HFD, a significantly smaller increase or even a decrease in T_1_ relaxation time was observed after eNOS inhibition with L-NAME. Surprisingly, the area under the ΔT_1_ − post-injection time curve tended to be smaller in HFD-fed animals even after only 3 days of HFD, and was significantly smaller after 7 days of HFD (Fig. 2F). The severity of coronary microvascular dysfunction, as assessed based on L-NAME response and cumulative ΔT_1_ (Fig. 2F), was similar in magnitude after 7 days, 2 weeks and 4 weeks of HFD, but further deteriorated with a HFD duration of 8 weeks.

### Coronary microvascular dysfunction in mice fed a high-fat diet for 1–8 weeks

**Figure 3:**
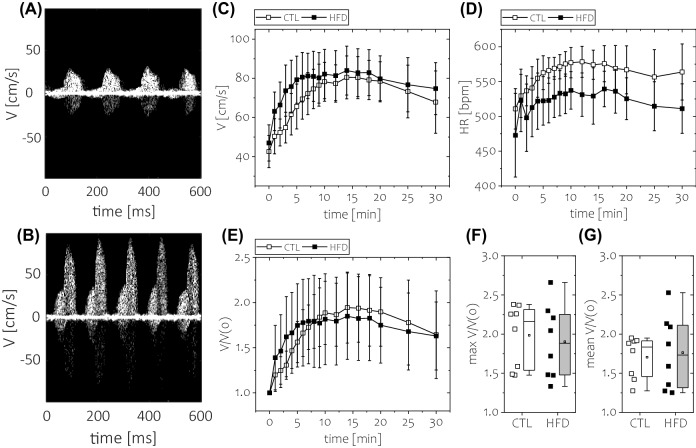
Assessment of in vivo coronary artery flow reserve with Doppler flow velocity measurements. Representative examples of coronary blood flow velocity speckles (**A**) at baseline and (**B**) 12 min after 1 mg/kg regadenoson injection. In each case, the speckle waveform was recorded for a fixed time of 800 ms. Note the increased number of waveforms recorded after vasodilation compared to the rest condition due to an increased heart rate. Time course of (**C**) absolute blood flow velocity, (**D**) heart rate and (**E**) flow reserve recorded dynamically after regadenoson injection up to 30 min post-injection. The quantification of the coronary reserve was performed with either (**F**) peak blood velocity reserve or (**G**) mean blood flow velocity reserve calculated between 6 and 18 min post-injection. For data in (**F**) and (**G**), results for control mice are given in white, whereas data for high-fat diet-fed mice are given in grey. The following number of animals was used n_CTL_ = 8, n_HFD_ = 8.

In order to verify whether coronary microvascular dysfunction in mice fed a HFD was associated with the impairment of coronary flow reserve, the vasodilation response of the coronary artery to regadenoson (1 mg/kg) was probed with Doppler flow velocity mapping in control and HFD-fed animals after 7 days on either diet. Examples of blood flow speckles recorded at rest and during vasodilation induced by regadenoson are shown in Fig. 3A,B, respectively. Significantly increased blood flow velocity was obtained after regadenoson injection (Fig. 3C,E). This was accompanied by an increase in heart rate during the vasodilation protocol (Fig. 3D). No significant differences in either peak coronary blood flow reserve (Fig. 3F) or mean blood flow reserve (Fig. 3G) were found between control and HFD-fed animals.

### Histological evaluation of ex vivo heart tissue samples in mice fed a high-fat diet for 1–8 weeks

**Figure 4:**
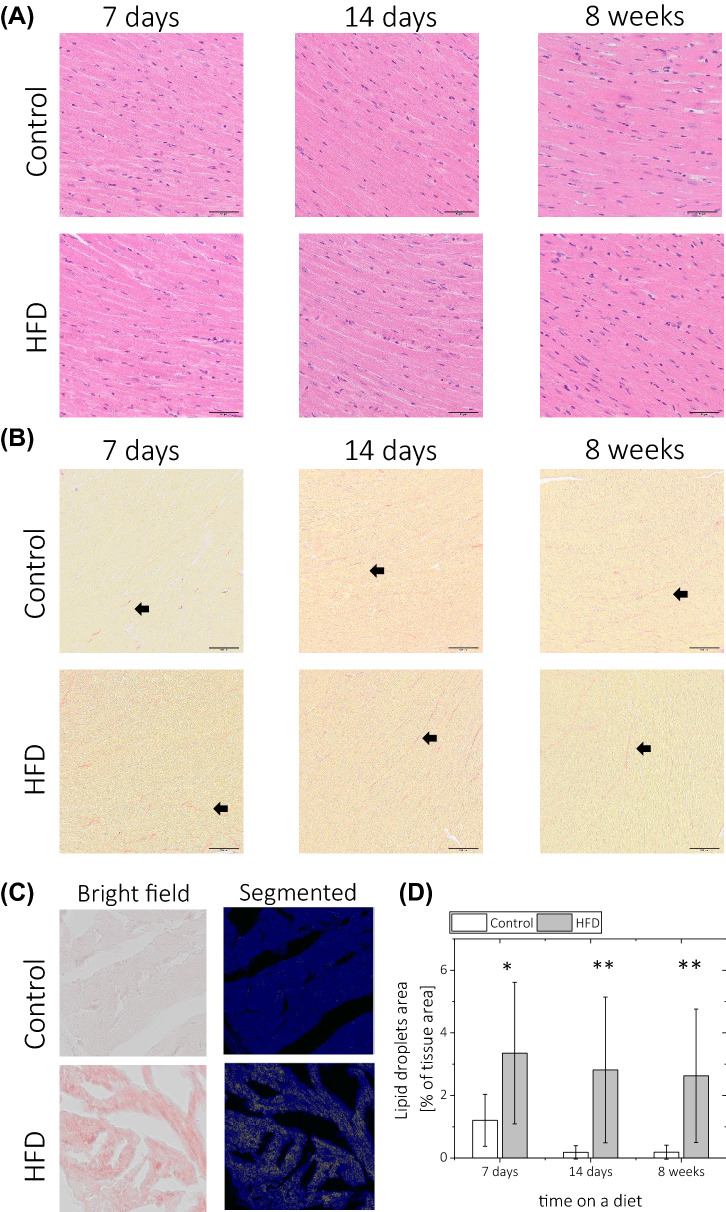
Histological evaluation of ex vivo heart samples obtained at 1, 2 and 8 weeks on a control and HFD diet. Examples of (**A**) haematoxylin and eosin staining and (**B**) Picrosirius red (PSR) staining (black arrow points towards collagen fibre). (**C**) Examples of native, bright-field image of Oil Red O (ORO)-stained sections and corresponding segmented images for a sample collected after 8 weeks of a diet. (**D**) Quantitative analysis of lipid droplets in ORO-stained section calculated with segmented images as shown in (**C**). The percentage of the area with lipid droplets was obtained as a ratio of lipid content area to total tissue sample area. The following number of animals was used n_CTL_ = 5, n_HFD_ = 5 for each time point for diet duration.

**Figure 5:**
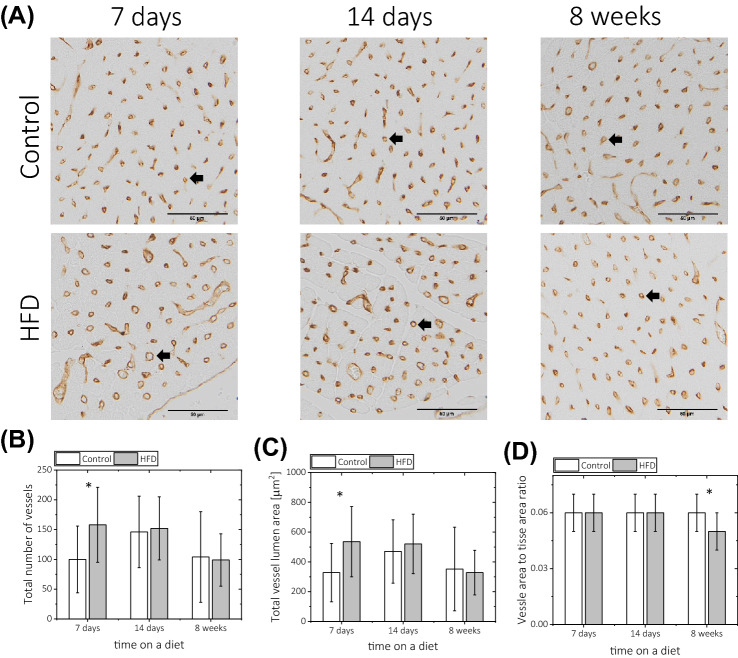
Effects of the high-fat diet on coronary microvascular capillary morphology as assessed by ex vivo lectin-based immunohistochemical staining. (**A**) Examples of stained bright-field images obtained at 1, 2 and 8 weeks on a diet. The black arrow points toward a perpendicular cross-section of the vessel lumen. (**B**) Total number of vessels receded in the whole field of view, (**C**) total vessel lumen (of only vessels placed perpendicular to the imaging field) and (**D**) the ratio of vessel lumen area to tissue area. The following number of animals was used n_CTL_ = 5, n_HFD_ = 5 for each diet duration time point.

Standard histological evaluation of cardiac sections obtained from mice at 1, 2 and 8 weeks of HFD did not reveal any microscopic alterations in HFD-fed animals compared to controls (Fig. 4A). There was also no difference in collagen deposition between control and HFD-fed animals (Fig. 4B). However, HFD induced mild but significant myocardial steatosis (4.5–4.8%), which was persistent throughout the duration of the diet (Fig. 4C,D). Interestingly, 1 week of HFD feeding resulted in increases in the total number of microvascular vessels and the total vessel lumen area (Fig. 5A–C), as assessed by IHC staining with lectin. This effect was not observed after 2–8 weeks of HFD feeding. In contrast, the ratio of vessel lumen to tissue area was decreased after 8 weeks of HFD feeding, as shown in Fig. 5D.

### Left ventricle global function in mice fed a high-fat diet for 1–8 weeks

**Figure 6:**
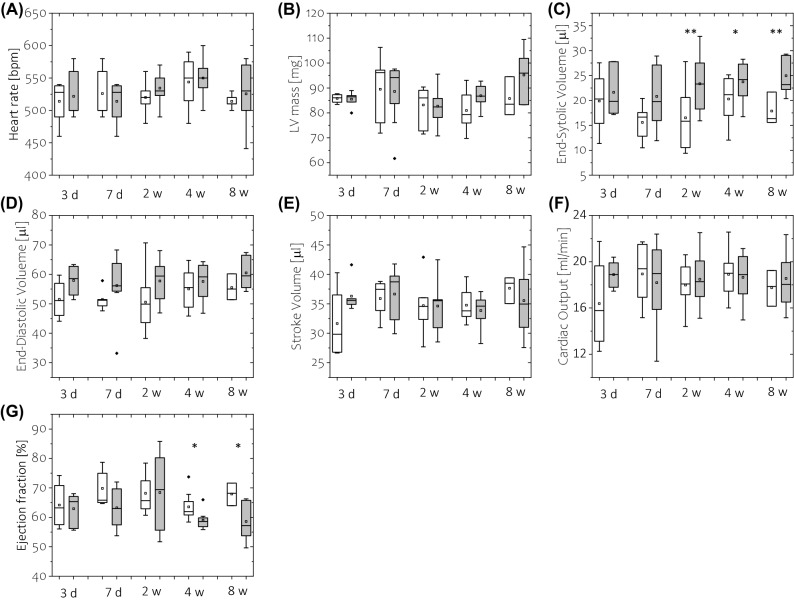
Estimates of LV global function during diet duration. (**A**) Heart rate, (**B**) LV mass, (**C**) End-Systolic Volume, (**D**) End-Diastolic Volume, (**E**) Stroke Volume, (**F**) Cardiac Output, and (**G**) Ejection Fraction obtained from LV volume CINE imaging segmentation. The white -control group, grey—HFD group. The following number of animals was used: measurements at diet time *t* = 3 days, n_CTL_ = 5, n_HFD_ = 6; *t* = 7 days n_CTL_ = 5, n_HFD_ = 8;* t* = 2 weeks n_CTL_ = 6, n_HFD_ = 9;* t* = 4 weeks n_CTL_ = 8, n_HFD_ = 8; *t* = 8 weeks n_CTL_ = 5, n_HFD_ = 7.

**Figure 7:**
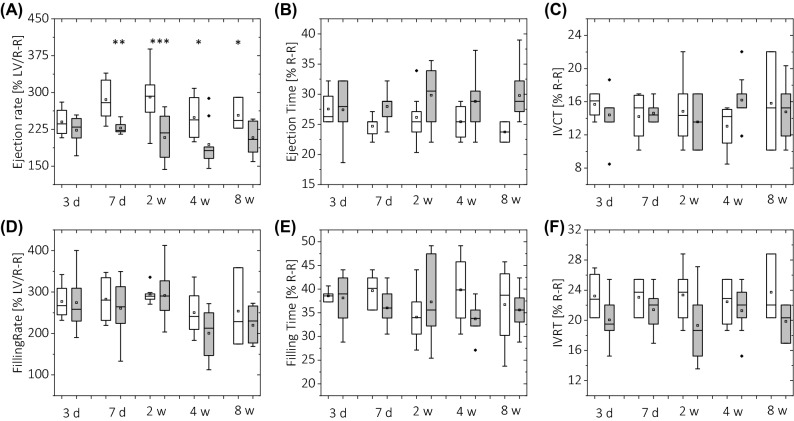
Assessment of LV systolic (**A**–**C**) and diastolic (**D**–**F**) function based on LV volume-time curve analysis obtained in control, standard diet-fed mice (white) and high-fat diet-fed mice (grey). The LV function was quantified using LV cavity ejection (**A**) and filling (**D**) rates, ejection and filling times (**B**,**E**) and isovolumetric contraction (**C**) and relaxation (**F**) times. Data in (**B**), (**C**), (**E**) and (**F**) are expressed as a percentage of normalised cardiac cycle length. The following number of animals was used: measurements at diet time *t* = 3 days, n_CTL_ = 5, n_HFD_ = 6; *t* = 7 days n_CTL_ = 5, n_HFD_ = 8;* t* = 2 weeks n_CTL_ = 6, n_HFD_ = 9;* t* = 4 weeks n_CTL_ = 8, n_HFD_ = 8; *t* = 8 weeks n_CTL_ = 5, n_HFD_ = 7.

Global LV functional analysis is presented in Fig. 6. No significant difference in heart rate was found between the control and HFD-fed animal at any time point from 3 days to 8 weeks. Similarly, no difference in LV mass was found; however, a tendency toward an increase in LV mass was observed after 8 weeks on a HFD (p = 0.08). Stroke volume and cardiac output were preserved in HFD mice compared to control mice. Ejection fraction was not reduced after up to 2 weeks of HFD feeding, but was reduced after 4 and 8 weeks of HFD feeding compared with control animals. A reduction in ejection rate was observed after only 7 days in HFD-fed mice compared to the control group (Fig. 7A).

**Figure 8:**
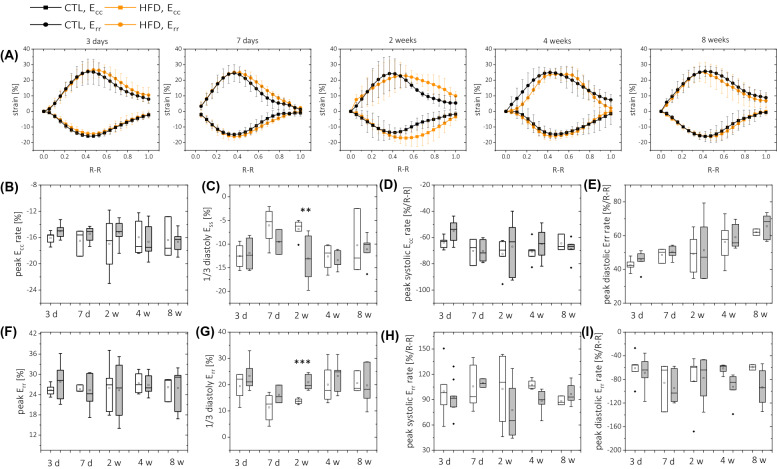
Left ventricle circumferential E_cc_ (**B**–**E**) and radial E_rr_ (**F**–**I**) deformation assessment with strain MR tagging analysis obtained for control animals (white) and HFD-fed animals (grey). (**A**) E_cc_/E_rr_ strain curves recorded for all animals. Maximum deformation (peak E_cc_/E_rr_—**B**,**F**) and respective peak strain rates at systole (**D**,**H**) and diastole (**E**,**I**) were calculated from strain–time curves averaged over eight myocardial sectors. For evaluation of strain values in diastole, strain values at 1/3 of diastole were taken (**C**,**G**). Strain rates were calculated with respect to normalised cardiac cycle length. The following number of animals was used: measurements at diet time *t* = 3 days, n_CTL_ = 5, n_HFD_ = 6; *t* = 7 days n_CTL_ = 5, n_HFD_ = 8;* t* = 2 weeks n_CTL_ = 6, n_HFD_ = 9;* t* = 4 weeks n_CTL_ = 8, n_HFD_ = 8; *t* = 8 weeks n_CTL_ = 5, n_HFD_ = 7.

There was no difference in ejection time or isovolumetric contraction time between the two groups (Fig. 7B,C). Similarly, there was no difference in filling rate or filling time (Fig. 7D,E). Isovolumetric relaxation time was reduced in all HFD-fed groups compared to control animals, but these results were not statistically significant (Fig. 7F). End-diastolic volume tended toward an increase at 3 and 7 days on a HFD, while end-systolic volume significantly increased at 2–8 weeks on a HFD. Both circumferential (E_cc_) and radial (E_rr_) peak strain values were preserved in HFD-fed mice compared to standard diet-fed animals. Similarly, no difference was found in systolic strain rates (systolic/diastolic SR_cc,_ systolic/diastolic SR_rr_). However, as shown in Fig. 8, significant changes in diastolic strain values were found at 2 weeks on a HFD, with reduced diastolic R_cc_ and reduced diastolic R_rr_ values.

## Discussion

In this study, taking advantage of an MRI-based method, we demonstrated for the first time to our knowledge that coronary microvascular dysfunction is a very early phenomenon in mice fed a HFD and was present after as short as 7 days on a HFD. Importantly, coronary microvascular dysfunction induced by short-term feeding with HFD was not associated with impaired coronary reserve or with impaired capillary density and occurred prior to systemic insulin resistance and alterations in diastolic and systolic cardiac function induced by HFD in mice. These findings highlight the relative vulnerability of coronary microvasculature to HFD, but also indicate that early development of impaired function of coronary microvasculature may contribute to the cardiomyopathic phenotype in metabolic diseases and thus represent an important therapeutic target^2^.

In this study, a multiparametric CMR-based in vivo approach was used to comprehensively describe coronary microcirculation functional status as well as cardiac global function. In particular, coronary microcirculation functional status was directly probed in vivo by measuring T_1_ relaxation proportional to eNOS function^18^, while the assessment of heart function was based not only on the left ventricle systolic/diastolic volume, but also using a left ventricle volume–time curve and left ventricle strain analysis, an approach that has previously been shown to be sensitive to early changes during HFD^22,23^.

The major finding of this study was that the coronary microvascular endothelial phenotype was already compromised in pre-insulin-resistant animals. Significant impairment of NO-dependent function was observed after 7 days of HFD, and a tendency could already be seen as short period as after 3 days on a diet.

In this study, decrease (in respect to baseline) in endothelial permeability upon L-NAME injection as assessed based on T1 mapping was observed for animals fed 2–8 weeks with HFD as compared with control animals, while in previous report by Cui et al.^24^ T_1_ also increased in response to LNAME in normal mice but remained unchanged in HFD mice. This difference could be ascribed to severity of eNOS dysfunction and dose of L-NAME used. While short treatment with HFD (3 days, 2–8 weeks) resulted in a moderate coronary endothelial dysfunction, observed as diminished endothelial permeability after L-NAME injection, prolonged diet duration (12–18 weeks) might have led to more severe coronary endothelial dysfunction. Furthermore, in our study higher dose of L-NAME was used (100 vs 4 mg/kg), suggesting more efficient NOS inhibition in vivo, that might have translate into more sensitive detection of residual NO-dependent function in coronary circulation of HFD mice.

Previous studies^18^ that employed eNOS inhibition with L-NAME also observed that changes in endothelial function status precede the changes in myocardial blood flow reserve^18^. However, these changes occurred after a much longer time on the diet, at week 12 (eNOS dysfunction) and week 18 (perfusion reserve), and early time points were not studied. In this study early changes in NO-dependent function did not coincide with impaired coronary reserve that was fully preserved. The preserved myocardial blood flow reserve found in this study after t = 2 weeks of HFD could be explained by the fact that, unlike in humans, in rodents adenosine A2 receptors mediate vasorelaxation only in part through endothelium^25^. Moreover, eNOS depletion was shown to trigger compensatory mechanism via nNOS and prostacyclin production that preserve adenosine mediated response in coronary flow^26^. Finally, changes in endothelial permeability could be altered even though endothelium-dependent vasodilation mediated by NO, e.g. induced by flow (flow-mediated dilation, FMD) was fully preserved^27^.

In addition, a previous report^8^ using isolated coronary arteries from mice fed HFD demonstrated increased vasoconstriction and reduced vasodilation responses to acetylcholine infusion compared with control mice, accompanied by decreased levels of NO and prostacyclin after 8 weeks of HFD feeding, and number of studies demonstrated coronary endothelial dysfunction after long-term HFD^10^. Overall, the mechanisms involved in the impairment of endothelial function in the coronary artery were ascribed to an increase in oxidative stress^28–30^ and subsequent eNOS uncoupling, the major hallmark of impaired NO-dependent function of coronary circulation^31^ associated with changes in proinflammatory cytokines and adipose tissue-derived adiponectin levels, increased free fatty acid levels and altered cellular signalling^32^. Furthermore, long-term feeding with HFD was shown to induce significant interstitial fibrosis^33^, resulting in extracellular matrix remodelling and stiffening of the vessels, including coronary arterioles^34^. However, in our study, no significant fibrosis was found; moreover, cardiac histology and myocardial capillary density were not significantly altered after up to 8 weeks of HFD and coronary microvascular dysfunction was detected prior to systemic insulin resistance. Furthermore, the early coronary microvascular dysfunction was not accompanied by impairment in coronary flow reserve. A decrease in myocardial perfusion capacity was previously reported for rats kept on a HFD for 6 weeks^35^, but was not observed in mice until week 18 on a HFD diet^34^.

Altogether, our results support the hypothesis that compromised NO-dependent function in coronary microcirculation is the driving force behind the early coronary microvascular dysfunction^36,37^. In this study, early coronary microvascular dysfunction was associated with a significant myocardial lipid accumulation that was observed for the duration of the HFD. Other studies of HFD also reported myocardial metabolic lipid toxicity^38^ and lipid tissue content remodelling^22^, suggesting that excessive lipid accumulation might contribute to early coronary microvascular dysfunction and subsequent alterations in cardiac function. Given the primary role of endothelia as gatekeepers of cardiac metabolism^39^, it is tempting to speculate that coronary microvascular dysfunction contributes to cardiac metabolic alterations and to functional impairment in cardiac function as a result of dysfunctional delivery of nutrients and hormones to cardiac cells^39^.

Although we did not study a possible mechanistic link between coronary microvascular dysfunction and cardiac dysfunction here, we demonstrated clearly the temporal relationship between these two phenomena. To the best our knowledge, our study was the first to unequivocally demonstrate in vivo that early endothelial dysfunction in coronary microcirculation preceded alteration in cardiac function in mice fed a HFD. Indeed, early endothelial dysfunction in coronary microcirculation was already detected after 7 days of HFD, yet global heart function, as assessed by measuring the change in ejection fraction, was compromised later in the diet duration, starting at 4 weeks.

Multiple studies^40^ have described the effect of a HFD on cardiac function in mice, with a consensus that long-term HFD leads to cardiac hypertrophy^41^, fibrosis^42^, diastolic dysfunction^38^ and impairment in coronary flow reserve^34^, though the extent of the impairment of systolic cardiac function depended on the exact diet composition and diet duration^9,23,43^. Indeed, conflicting results regarding early cardiac dysfunction in HFD-fed animals exist in the literature^9,44–46^. In some reports, mice fed short-term (5 weeks) on a HFD showed^22^ early signs of reduced myocardial contractility, similar to the data presented in our study. On the other hand, in most of the studies, diastolic dysfunction was detected only after long-term HFD^38^. In our work, subtle alterations in diastolic circumferential and radial strain values were found after as short as 2 weeks of HFD. Interestingly, signs of diastolic dysfunction faded thereafter with HFD feeding, suggesting early triggering of compensatory mechanisms^47^. In our study, a standard 60% HFD was supplemented with 1% cholesterol, which could have accelerated the detrimental effects of coronary microvascular dysfunction and diastolic cardiac dysfunction^12,48^.

Interestingly, it has recently been demonstrated that accelerated impairment of cardiac function during HFD could be obtained using the so-called “double hit” approach, in which matured animals (aged 16–20 months) are exposed to either HFD alone or HFD + L-NAME^49^/Angiotensin II^50^. Such combinations allowed the development of a murine model of diastolic heart failure that closely resembles the clinical description of heart failure with preserved ejection fraction, but still requires a prolonged HFD duration (15–20 weeks). Using our MRI-based methodology, we were able to detect coronary microvascular dysfunction and subsequent alterations in cardiac function with short-term HFD (60% HFD + 1% cholesterol) feeding, offering an attractive alternative methodological approach to study the effectiveness of novel therapeutic strategies to mitigate coronary microvascular dysfunction and subsequent cardiac dysfunction^51,52^.

Considering that eNOS plays a crucial role in sustaining microvascular endothelial homeostasis and contributes to vasoprotection, inhibition of platelet aggregation and leukocyte adhesion, in vivo monitoring of eNOS function might be indispensable in the further development of basic and clinical research for effective endothelium-guided therapy^53,54^. A potential replacement for L-NAME injection is applying T_1_ mapping dynamically during vasodilation, in which increased blood flow stimulates NO production and can alter microvascular endothelial permeability^55^.

In conclusion, using MRI-based methodology in vivo, we demonstrated that short-term feeding with a HFD triggers early coronary microvascular dysfunction that precedes changes in global heart function, emphasising the high vulnerability of coronary microvasculature to HFD-induced insult. Therefore, coronary microvasculature constitutes the *locus minoris resistantiae* of the cardiometabolic diseases that require targeted therapeutic interventions.

## Conclusion

Short-term (3 days–8 weeks) feeding with a HFD triggers early impairment of endothelial microvascular status and precedes changes in global heart function.

## Methods

### Animal handling

A total of 48 C57BL/6 mice (10–14-week-old males weighing 25–35 g, obtained from Mossakowski Medical Research Centre, Polish Academy of Sciences, Warsaw, Poland) were included in this study. The animals were randomly assigned to one of two experimental groups and fed either a HFD (60% kcal from fat + 1% cholesterol; ZooLab, Krakow, Poland) or a control diet (AIN93G). The animals were kept on the diet for 3, 7, 14, 28 or 56 days. The size of each experimental group is reported in the legends of the corresponding graphs. The animals were housed under a 12 h light/dark cycle in pathogen-free conditions with ad libitum access to food and water. All animal experiments were performed in adherence with the Local Ethics Committee of Jagiellonian University (Krakow, Poland) and in accordance with the Guide for the Care and Use of Laboratory Animals of the National Academy of Sciences (NIH publication No. 85–23, revised 1996), as well as the Guidelines for Animal Care and Treatment of the European Community. All results concerning experiments on animals follows the recommendations in the ARRIVE guidelines.

### Glucose tolerance test

Mice were fasted for 4 h with access to water. Glucose levels were measured using a standard glucometer in a drop of blood from the tail, cut at the top. The measurements were taken before and after 15, 30, 45, 60 and 120 min of intraperitoneal glucose administration (2 g/kg b.w.). For quantification, area under the blood glucose concentration–time curve (AUC) was calculated.

### Magnetic resonance imaging

For imaging, the animals were placed in a prone position, with the heart in the centre of the detection coil. Anaesthesia was delivered via a nose cone at a constant level of 1.75% isoflurane in a mixture of 0.4 l/min O_2_: 0.8 l/min air. Body temperature was monitored with an endorectal probe and maintained in the range of 35.5–36.5 °C. All MR experiments were recorded with a 9.4 T small animal MRI scanner (Bruker BioSpec, Ettlingen, Germany) equipped with a 1000 mT/m gradient coil with a maximum slew rate of 3500 T/m/s. A 36 mm quadrature volume coil was used for RF excitation and detection. To evaluate the global function of LV, the bright-blood cine images were collated in 6–7 contiguous slices covering the whole ventricle volume using a flow-compensated, prospectively gated gradient-echo FLASH sequence with the following parameters: FOV 30 × 30 mm^2^, acquisition matrix: 192 × 192, TE/TR = 2.3/5 ms, slice thickness = 1 mm, number of averages = 4, flip angle  =  11°.  Depending on the heart rate, between 22 and 24 cine frames were acquired. The filling and ejection rates of LV were obtained with a high-frame-rate, retrospectively gated cine FLASH sequence (IgFLASH) in a mid-ventricular, short-axis slice. The following acquisition parameters were used: FOV 30 × 30 mm, acquisition matrix 128 × 128, TE/TR = 1.3/4.2 ms, slice thickness = 1 mm, number of repetitions = 1200, flip angle = 11°. The data were reconstructed to 60 frames per cardiac cycle using a vendor-provided macro (ParaVision 6.0.1, Bruker BioSpin, Ettlingen, Germany). Tagged cine images were obtained using a double-gated FLASH sequence (TE/TR 1.5/4.8 ms, flip angle 11°, FOV 30 × 30  mm^2^, matrix 192 × 192, slice thickness 1.0 mm, 16 repetitions, 20–25 frames) with a spatial modulation of magnetization (SPAMM) module for tag generation (square tags: line thickness 0.2 mm, span 0.6 mm).

Myocardial T_1_ mapping was performed with a variable flip angle (VFA)^56^ before and after *i.v.* injection of 100 mg/kg Nω-Nitro-l-arginine methyl ester hydrochloride (L-NAME, Sigma Aldrich, Poznan, Poland). The pre-injection map was obtained with five RF flip angles of 2°, 5°, 8°, 11° and 13° and RF-spoiled retrospectively gated GRE FLASH sequence (IntraGate, Bruker Bio Spin, Ettlingen, Germany) with the following acquisition parameters: FOV 30 × 30 mm^2^, acquisition matrix: 192 × 128, TE/TR = 1.6/10 ms, partial echo = 75%, slice thickness = 1 mm, number of repetitions = 300, with the navigators signal derived from slice refocusing signal and reconstructed to six cardiac cycle frames. The total acquisition time for each RF flip angle was 4 min. The post-injection maps were obtained with a single RF flip angle of 13°, and imaging and T_1_ values were derived based on the single changes between the pre-/post-injection values and the pre-injection T_1_ map (as shown in Fig. 2A,B). An average over the whole myocardium was taken as the pre-injection T_1_ value.

### Coronary artery flow reserve assessment

A separate cohort of animals was used for coronary artery blood flow velocity mapping during rest and maximal vasodilation. A Doppler Flow Velocity System (Indus Instruments, Texas, USA) equipped with a single transceiver 20 MHz Doppler probe was used. Anaesthesia was delivered via a nose cone at a level of 1.5–1.25% isoflurane in a mixture of 40% O_2_:80% air. The animals were placed in a supine position on a heating pad and secured to a four-channel ECG system with tape on each paw. An endorectal probe was inserted for continuous body temperature monitoring. A cannula was placed into a tail vein for injection of a vasodilator (1 mg/kg regadenoson). The upper chest was shaved with an electric razor and ultrasound gel was applied to both the chest and the Doppler probe. The probe was manually adjusted and secured with a micromanipulator. The correct position of the probe along the coronary artery was confirmed with a visual inspection of the blood flow velocity waveform and its relation to the ECG signal^57^. At each time point, blood flow velocity was recorded for 2000 ms, comprising 16–20 blood flow speckles. Speckles recorded during expiration were included in the analysis, and the average overall speckles were taken at the maximum value of blood flow velocity. The measurements were recorded before and 1, 2, 3, 4, 5, 6, 7, 8, 9, 10, 12, 14, 16, 18, 20, 25 and 40 min post-injection. Coronary flow reserve was calculated as the ratio between blood flow velocity after regadenoson injection to a pre-injection value. Each animal received a single injection with a constant volume of 100 µl.

### Data reconstruction and analysis

A time-volume curve (TVC) was calculated from LV volumes (including papillary muscles) assessed using short-axis slice-by-slice semiautomatic segmentation (Segment; Medviso). End-systolic (ESV) and end-diastolic (EDV) volumes, stroke volume (SV), ejection fraction (EF), cardiac output (CO) and cardiac index [CI x CO/body surface area (BSA); BSA = 9.822 × (body weight), ^2/3^] were assessed from the TVC. A piecewise linear regression (PLR) implemented in MATLAB 2021a (MathWorks), as described previously^58,59^ was used to obtain ejection (ER) and filling rates (FR), with slopes of segments fitted by PLR normalised to the individual SV and RR intervals. Durations of ejection (ET), isovolumic relaxation (IVRT), filling (FT) and isovolumic contraction phases (IVCT) were taken from the PLR model and normalised to the RR interval. For tagged images analysis, image registration^62^ was performed using a dedicated strain analysis plug-in using the freely available software Segment (Medviso, http://segment.heiberg.se)^61^. The midlevel strain maps were used to compute radial (E_rr_) and circumferential (E_cc_) strains (peak strain). Strains were assessed in eight consistent segments encircling the myocardial cross-section and then averaged.

Quantification of T_1_ changes in response to NOS inhibition was obtained as the area under the curve of ΔT_1_ – post-injection time (cumulative ΔT_1_).

Doppler flow velocity data were analysed using vendor-provided software (Doppler Signal Processing, Indus Instruments, Texas, USA), with a peak blood velocity of 12–16 speckles averaged for each time point.

### Histological analysis of myocardial tissue samples

Formalin-fixed and paraffin-embedded or 30% sucrose-fixed heart samples were used for histological analysis. Haematoxylin and eosin (H&E) staining was used for microscopic anatomy differentiation, Picrosirius red (PSR) was used for collagen imaging and immunohistochemical (HC) staining with lectin was used for the assessment of capillary density, as described previously^13,60^. For cardiomyocyte steatosis, myocardial sections were stained with Oil Red O (ORO). Quantitative analysis of stained images was obtained via image segmentation adopted in Ilastik (developed by the Ilastik team, with the partial financial support of the Heidelberg Collaboratory for Image Processing, HHMI Janelia Farm Research Campus and CellNetworks Excellence Cluster).

### Statistical analysis

All data are expressed as mean ± standard deviation (SD). Data from animals fed with a control diet or HFD for a given time were compared using a t-test for independent groups. The normality of distribution and homogeneity of variance were tested using the Shapiro–Wilk and Brown–Forsythe tests, respectively. If these assumptions were not fulfilled, the nonparametric Mann–Whitney U-test was performed. Results were considered statistically significant at p < 0.05, with the following notation used: *p < 0.05, **p < 0.01, ***p < 0.001.

## Abbreviations

HFD: High fat diet
IHD: Ischemic heart disease
CMD: Coronary microcirculation disease
L-NAME: Nω-nitro-l-arginine methyl ester hydrochloride
LV: Left ventricle
ESV: End systolic volume
EDV: End diastolic volume
SV: Stroke volume
CO: Cardiac output
EF: Ejection fraction
