# Author Correction: MRI-based in vivo detection of coronary microvascular dysfunction before alterations in cardiac function induced by short-term high-fat diet in mice

**DOI:** 10.1038/s41598-021-99611-3

**Published:** 2021-10-05

**Authors:** Grzegorz Kwiatkowski, Anna Bar, Agnieszka Jasztal, Stefan Chłopicki

**Affiliations:** 1 Jagiellonian Centre for Experimental Therapeutics (JCET), Jagiellonian University, ul. Bobrzynskiego 14, 30‑348 Kraków, Poland; 2 Chair of Pharmacology, Faculty of Medicine, Jagiellonian University Medical College, Grzegorzecka 16, 31‑531 Kraków, Poland

Correction to: *Scientific Reports* 10.1038/s41598-021-98401-1, published online 23 September 2021

The Acknowledgements section in the original version of this Article was incomplete.

“This work was supported by Team Tech–Core Facility program of the FNP (Foundation for Polish Science) co‐financed by the European Union under the European Regional Development Fund (project No. POIR.04.04.00‐00‐5CAC/17–00 granted to S.C.) and partially by the Polish National Science Centre via the Sonata programme (grant no. 2020/39/D/NZ7/02593 to G.K.). Anna Bar acknowledges the START scholarship, awarded by the Foundation for Polish Science (Foundation for Polish Science, START2020 program).”

now reads:

“This work was supported by Team Tech–Core Facility program of the FNP (Foundation for Polish Science) co‐financed by the European Union under the European Regional Development Fund (project No. POIR.04.04.00‐00‐5CAC/17–00 granted to S.C.) and partially by the Polish National Science Centre via the Sonata programme (grant no. 2020/39/D/NZ7/02593 to G.K.). Anna Bar acknowledges the START scholarship, awarded by the Foundation for Polish Science (Foundation for Polish Science, START2020 program). The open-access publication of this article was funded by the Priority Research Area BioS under the program “Excellence Initiative – Research University” at the Jagiellonian University in Krakow.”

The original Article has been corrected.

